# Sub-elite sprinters and rugby players possess different morphological characteristics of the individual hamstrings and quadriceps muscles

**DOI:** 10.1371/journal.pone.0259039

**Published:** 2021-10-26

**Authors:** Raki Kawama, Masamichi Okudaira, Tatsuya Shimasaki, Hirohiko Maemura, Satoru Tanigawa

**Affiliations:** 1 Graduate School of Health and Sports Sciences, Doshisha University, Kyoto, Japan; 2 Graduate School of Comprehensive Human Sciences, University of Tsukuba, Ibaraki, Japan; 3 Institute of Health and Sports Sciences, University of Tsukuba, Ibaraki, Japan; Universite de Nantes, FRANCE

## Abstract

Numerous studies have clarified that sprinters possess unique morphological characteristics of the thigh muscles compared with non-athletes. However, little evidence is available regarding the morphological differences between sprinters and rugby players. This study aimed to examine the morphological differences in the individual hamstrings and quadriceps femoris muscles between sub-elite sprinters and rugby players. Ultrasound images were acquired from the proximal, middle, and distal regions of the thigh. From the images, the anatomical cross-sectional areas were calculated for 14 sub-elite sprinters, 14 rugby players, and 14 non-athletes. The calculated anatomical cross-sectional areas were normalized to two-thirds power of the body mass, and the normalized values of all regions were averaged as those of the individual muscles. In the hamstrings, the sizes of the biceps femoris short head and semitendinosus were greater in the sprinters than in the rugby players and/or non-athletes (all *p* < 0.05). In contrast, in the quadriceps femoris, the sizes of the rectus femoris, vastus lateralis, and vastus intermedius were the greatest in the rugby players (all *p* < 0.05). In the middle region of the biceps femoris short head and the proximal-middle regions of the semitendinosus, the muscle sizes were greater in the sprinters than in the rugby players (all *p* < 0.05), and vice versa in the middle-distal regions of the rectus femoris (all *p* < 0.05). These results suggest that 1) sub-elite sprinters possess larger sizes of the biceps femoris short head and semitendinosus, whereas rugby players have larger sizes of the rectus femoris, vastus lateralis, and vastus intermedius, and 2) each of the athletes has different size distributions, especially along the lengths of BFsh, ST, and RF. The findings of the present study would be helpful for rugby players in designing training regimens aimed at enhancing sprint performance.

## Introduction

Sprint running is a key component of performance in rugby [1,2]. For the rugby players, sprinters would be recognized as an ideal model to enhance sprint performance because they can generate a much faster running speed than other athletes [3]. It has been considered that the impressive running speed of sprinters is mainly achieved by the great joint moment of the lower limb [4]. Theoretically, the joint moment is generated by the muscle force, and the maximum muscle force is strongly associated with muscle size [5]. Thus, numerous studies have examined the morphological characteristics of sprinters to identify the muscles that are most important for achieving a high running sprint performance [4,6–8]. For players and coaches in rugby, understanding the gap in the muscle morphologies between the rugby players and sprinters could help them to set a clear goal and to develop a purposeful training regimen. However, there is little information about the morphological differences between sprinters and rugby players; the muscle morphologies of sprinters have generally been compared with those of non-athletes in most studies [4,6–8].

It has been suggested that sprinters have a larger size of a specific muscle than the non-athletes [4,6–8]. For example, the magnitude of difference in the relative muscle volume between sprinters and non-athletes was the greatest in the semitendinosus (ST) among the hamstring muscles [6,8]. Moreover, a significant difference in muscle size between both groups was found only in the rectus femoris (RF) among the quadriceps femoris muscles [4]. These observations suggest that specific muscles such as ST and RF may play an important role in maximal speed sprinting. Because sprinters usually repeat sprint at a much faster speed than rugby players [3], the aforementioned muscles may be more hypertrophied in sprinters than in rugby players. Despite this possibility, it remains unknown whether the muscle sizes in the individual hamstrings and quadriceps femoris muscles differ between sprinters and rugby players. Furthermore, it has been demonstrated that the magnitude of muscle hypertrophy was non-uniform along the length of the quadriceps femoris muscles in Olympic weightlifters [9] and cyclists [10] who repeatedly perform their competitive movements. The non-uniform muscle hypertrophy was also observed in a recent study that examined the size distribution within the hip extensor muscles in sub-elite sprinters [8]. These findings provide a possibility that repeated competitive movements lead to movement-specific muscle size distribution along its length. However, little is known about whether sprinters and rugby players have different muscle size distributions along the length of the individual thigh muscles. Knowing such a morphological difference would be valuable for rugby players to identify the muscles and regions that should be targeted to improve sprint performance in daily training.

This study aimed to investigate the morphological differences in the individual hamstrings and quadriceps femoris muscles between sub-elite sprinters and rugby players. Based on previous findings [4,6,8], we hypothesized that muscle sizes of ST and RF are larger in sprinters than in rugby players. Also, it was postulated that sprinters and rugby players have different size distributions within the individual thigh muscles [8–10].

## Materials and methods

### Participants

Fourteen male sprinters, 14 male rugby players, and 14 male non-athletes volunteered for this study (Table 1). The sprinters and rugby players were recruited from sports club teams of our university while the non-athletes did not belong to those teams at the university. The number of participants in each group was calculated with a statistical power of 80%, an α error of 0.05, and an effect size of 0.50, using the G*power software (version 3.1.9.7). An effect size of 0.50 was assumed based on a previous study, which found a significant main effect of the participants’ group (i.e., sprinters and non-athletes) on the sizes of the individual posterior thigh muscles [8]. The power analysis indicated that the required total sample size was 42. Thus, 14 participants were recruited for each group in the present study. The personal best record of the sprinters ranged from 10.35 to 10.97 (10.71 ± 0.22) s for the 100-m sprint of sprinters, and the best record within three months before the experiment ranged from 10.49 to 11.20 (10.86 ± 0.24) s. The team of rugby players came third in the All-Japan University Rugby Football Championship 2 years ago. In addition, the rugby players could be divided into forwards (n = 7) and backs (n = 7) based on their competitive experiences. Nine of them, including players aged < 20 years, belonged to the Japanese national rugby team. The sprinters had normally engaged in five weekly training sessions (three sessions of sprint running and two sessions of resistance training), while the rugby players participated in six training sessions (four rugby sessions and two combined sessions of resistance training and sprint running). The non-athletes had not engaged in conventional sports activities or regular resistance training for at least two years before the experiment. None of the participants had a history of severe musculoskeletal injuries, neuromuscular or orthopedic diseases that result in >10 days lost from training and competition over the past two years. All participants were carefully informed of the purpose, procedures, and risks related to this study and they provided written informed consent before participation in this study. This study was approved by the research ethics committee of University of Tsukuba (no. 019–9) and was conducted in accordance with the Declaration of Helsinki.

**Table 1:** Anthropometrics and training profiles of the sub-elite sprinters (n = 14), rugby players (n = 14), and non-athletes (n = 14).

	Sprinters	Rugby players	Non-athletes
**Age (years)**	21.1 ± 1.6	20.6 ± 1.4	23.7 ± 0.8
**Height (cm)**	173.1 ± 5.9	176.7 ± 6.2	172.8 ± 5.7
**Body mass (kg)**	67.1 ± 5.4	89.8 ± 12.5	67.7 ± 7.1
**Years of sports experience (years)**	9.9 ± 2.1	11.9 ± 3.7	-

All data are presented as mean ± SD.

### Procedures

All experiments of the present study were conducted over two weeks (from August 17 to September 1, 2020) in our laboratory. To identify the measurement locations of anatomical cross-sectional area (ACSA), the thigh length (i.e., the distance between the greater trochanter and popliteal crease) was measured on the dominant leg preferentially used for ball kicking. The locations at 35%, 50%, and 65% of the thigh length were marked as an axial perpendicular line and defined as the proximal, middle, and distal regions, respectively. For ACSA measurements of the hamstrings, the participants were asked to lie prone on a massage bed at 0° of knee flexion. For ACSA measurements of the quadriceps femoris, the participants were instructed to lie supine on the bed. Consequently, the entire lower leg was placed in a box with 30° of knee flexion [11] because the lateral and medial sides of the quadriceps femoris were in contact with the bed at 0° of knee flexion. Ultrasound images were obtained from the proximal, middle, and distal regions of the thigh length using extended-field-of-view ultrasonography (EUP-L53, 6–10 MHz, 64 mm field of view, Noblus, Hitachi, Tokyo, JPN). The probe was moved slowly and carefully on the transverse plane perpendicular to the femur, from the medial to the lateral side of the thigh in each region after applying a transmission gel between the skin and the scanner. Then, a leather belt was attached to the thigh of the participant with minimal pressure to keep the probe perpendicular to the femur. All ultrasound measurements were performed by the same investigator with more than three years of experience in musculoskeletal ultrasonography.

**Fig 1:**
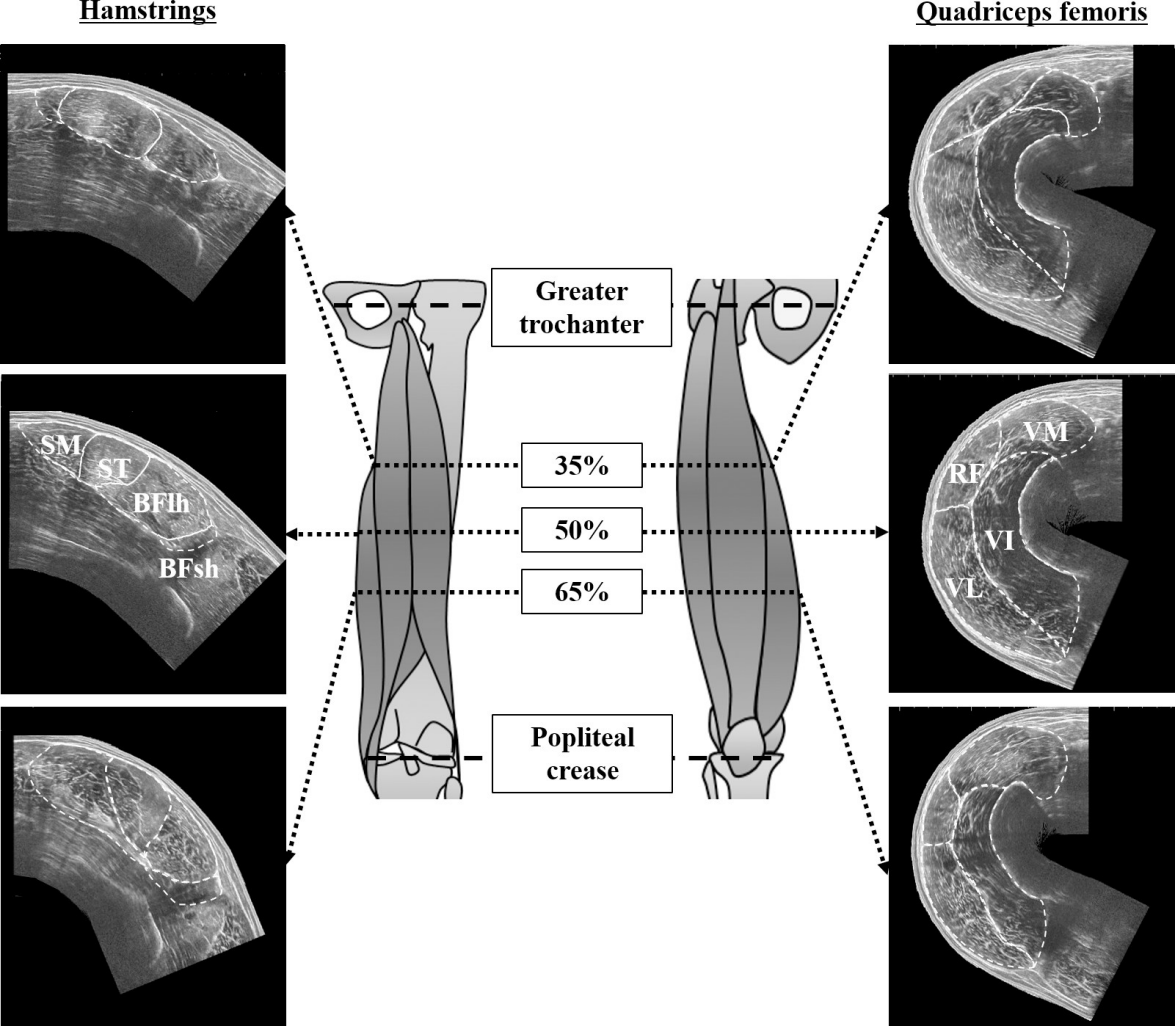
Measurement locations of the anatomical cross-sectional area and ultrasound images in the thigh muscles. The white broken line indicates the outline of the individual muscles of the hamstring and quadriceps femoris. BFlh, biceps femoris long head; BFsh, biceps femoris short head; ST, semitendinosus; SM, semimembranosus; RF, rectus femoris; VL, vastus lateralis; VM, vastus medialis; VI, vastus intermedius.

The ACSAs of the individual hamstrings (BFlh, biceps femoris long head; ST; and SM, semimembranosus) and quadriceps femoris muscles (RF; VL, vastus lateralis; VM, vastus medialis; and VI, vastus intermedius) were analyzed twice from the ultrasound images at the proximal, middle, and distal regions of the thigh using the Image J software (version 1.52, National Institutes of Health, Bethesda, MD, USA) (Fig 1). The ACSA of BFsh was calculated twice in only the middle and distal regions because the biceps femoris short head (BFsh) is a monoarticular muscle that does not cross the hip joint. The average of the two values was used for ACSA. It has been shown that ACSA is a function of the length to the second power, and body mass is a function of the length to the third power [12]. For this reason, the body mass could be theoretically presented as two-thirds of the power of body mass to convert it into the same dimension as ACSA. Thus, in the present study, ACSAs were normalized by the two-thirds power of the body mass (nACSA) to account for the influence of body mass on ACSA. This normalization procedure has been adopted in previous studies [8,13] and would allow us to compare the results between the present study and the previous studies. The nACSAs of the two or three regions were averaged as nACSAs of the individual muscles. Additionally, %differences in nACSA were calculated in the individual muscle groups, muscles, and regions to present the magnitude of difference in nACSA between the participant’ groups [8].

The measurement validity of extended-field-of-view ultrasonography has already been reported in previous studies [14,15]. However, we ensured test–retest reliability by acquiring two ultrasound images in each region of the thigh. The intraclass correlation coefficients and the coefficient of variation for ACSAs in the three regions of the individual hamstrings and quadriceps femoris were 0.902–0.999 and 0.2%–0.8%, respectively. The intraclass correlation coefficients could be interpreted as ‘almost perfect’ [16].

### Statistical analyses

All data are presented as means and standard deviations (SD). The Shapiro-Wilk normality test was adopted to examine the distribution of data on the nACSA of each region. The results showed that the data was non-Gaussian in nine regions (*p* = 0.001–0.037). Thus, the Kruskal-Wallis test was used to determine significant differences in the ACSA among the participants’ groups (sprinters, rugby players, and non-athletes) in each muscle group (hamstrings and quadriceps femoris), individual muscles (BFlh, BFsh, ST, SM, RF, VL, VM, and VI), and each region of the individual muscles (proximal, middle, and distal regions). If there was a significant difference among the participants’ groups, the Mann-Whitney U test was performed to identify which pairs indicated significant differences in the ACSA. The significance level was set at *p* < 0.05. This was modified using the Benjamini and Hochberg method for multiple tests [17]. The effect size (r) and its 95% confidence interval (95% CI) were calculated for the differences in nACSA between participants’ groups. All statistical analyses were performed using the SPSS software (version 25.0, IBM Corporation, Armonk, USA).

## Results

All absolute values of ACSA of the examined regions, muscles and muscle groups in the hamstrings and the quadriceps femoris are presented in S1 and S2 Tables.

**Fig 2:**
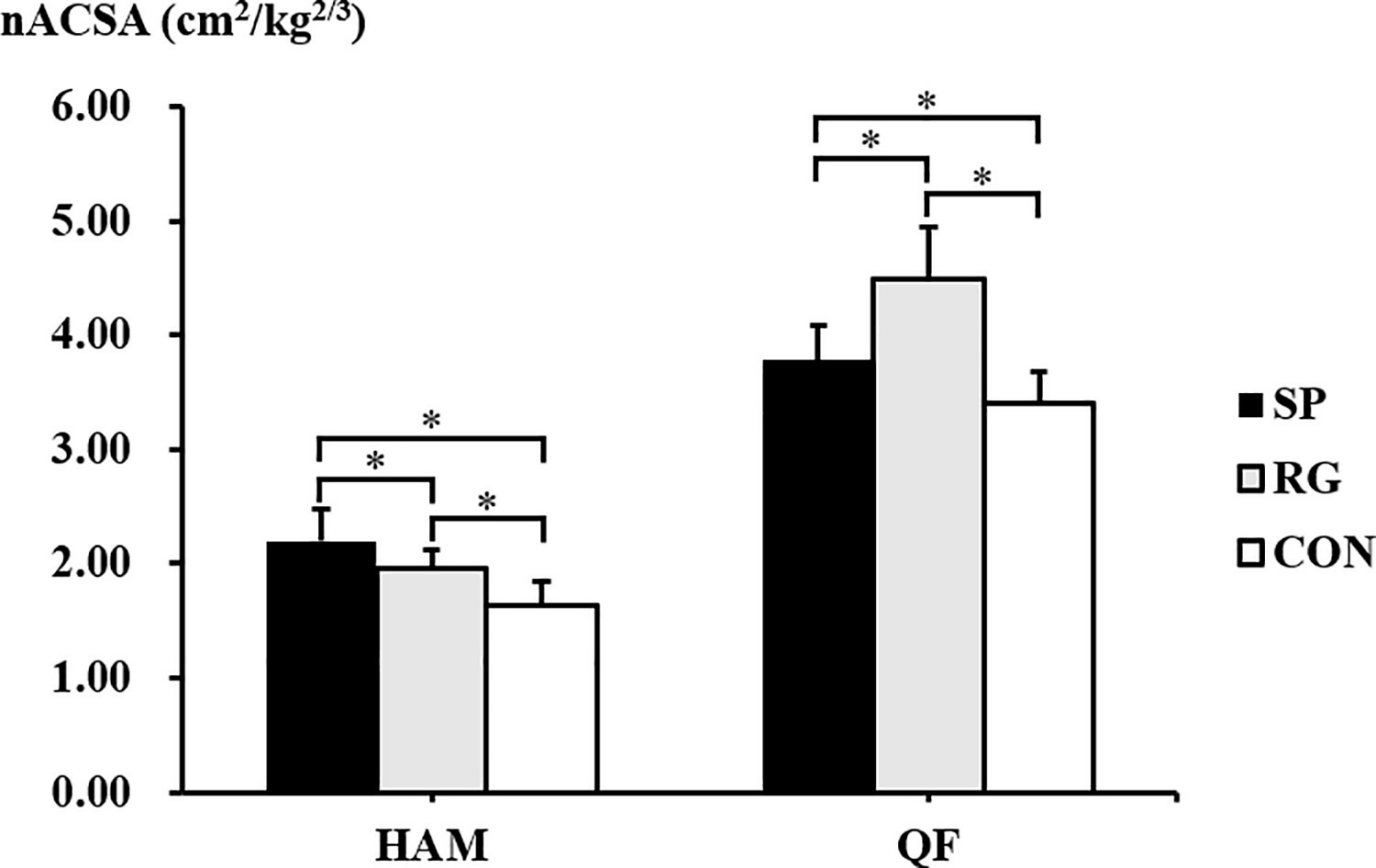
The normalized anatomical cross-sectional area of the hamstrings and quadriceps femoris in the sprinters (SP), rugby players (RG), and non-athletes (CON). *Significant difference between the groups. HAM, hamstrings; QF, quadriceps femoris.

The Kruskal-Wallis test revealed the significant differences in nACSA among the participants’ groups in the hamstrings and the quadriceps femoris (Fig 2). The nACSA of the hamstrings was significantly greater in the sprinters than in the rugby players (*p* < 0.001, r = 0.66, 95% CI = 0.45–0.80, %difference = 12.4%) and non-athletes (*p* < 0.001, r = 0.75, 95% CI = 0.52–0.88, %difference = 34.3%). The nACSA of the hamstrings in the rugby players was significantly greater than that in the non-athletes (*p* < 0.001, r = 0.65, 95% CI = 0.37–0.82, %difference = 19.6%). The nACSA of the quadriceps femoris was significantly greater in the rugby players than in the sprinters (*p* < 0.001, r = 0.69, 95% CI = 0.42–0.84, %difference = 15.9%) and non-athletes (*p* < 0.001, r = 0.79, 95% CI = 0.59–0.90, %difference = 32.2%). This nACSA was significantly greater in the sprinters than in the non-athletes (*p* = 0.003, r = 0.56, 95% CI = 0.24–0.77, %difference = 11.1%).

**Fig 3:**
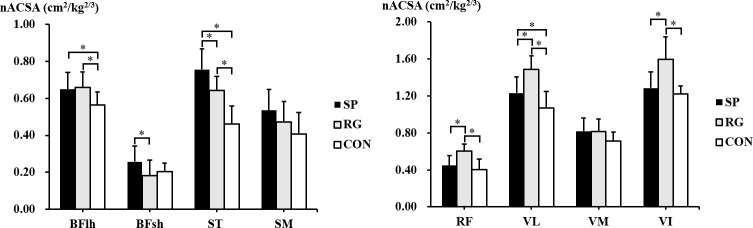
The normalized anatomical cross-sectional area of the individual muscles of the hamstrings and quadriceps femoris in the sprinters (SP), rugby players (RG), and non-athletes (CON). *Significant difference between the groups. BFlh, biceps femoris long head; BFsh, biceps femoris short head; ST, semitendinosus; SM, semimembranosus; RF, rectus femoris; VL, vastus lateralis; VM, vastus medialis; VI, vastus intermedius.

There were significant participants’ group differences in nACSA for BFlh (*p* = 0.012, r = 0.37, 95% CI = 0.09–0.62), BFsh (*p* = 0.011, r = 0.39, 95% CI = 0.10–0.62), and ST (*p* < 0.001, r = 0.74, 95% CI = 0.57–0.85), but not for SM (*p* = 0.055, r = 0.30, 95% CI = -0.01–0.55) (Fig 3). The nACSA of BFlh was significantly greater in the in the sprinters (*p* = 0.010, r = 0.49, 95% CI = 0.14–0.73, %difference = 15.3%) and rugby players (*p* = 0.012, r = 0.48, 95% CI = 0.13–0.72, %difference = 16.9%) than in the non-athletes. The nACSA of BFsh in the sprinters was significantly larger than that in the rugby players (*p* = 0.005, r = 0.53, 95% CI = 0.20–0.75, %difference = 41.0%). The nACSA of ST was significantly greater in the sprinters than in the rugby players (*p* = 0.004, r =, 95% CI = 0.21–0.76, %difference = 17.8%) and non-athletes (*p* < 0.001, r = 0.80, 95% CI = 0.61–0.90, %difference = 63.0%); it was also significantly greater in the rugby players than in the non-athletes (*p* < 0.001, r = 0.76, 95% CI = 0.53–0.88, %difference = 38.9%). The significant participants’ group differences in nACSA were found in RF (*p* < 0.001, r = 0.59, 95% CI = 0.35–0.76), VL (*p* < 0.001, r = 0.67, 95% CI = 0.46–0.81), and VI (*p* < 0.001, r = 0.56, 95% CI = 0.32–0.74) whereas that was not observed in VM (*p* = 0.052, r = 0.30, 95% CI = -0.01–0.55). In RF, nACSA was significantly greater in the rugby players than in the sprinters (*p* = 0.001, r = 0.63, 95% CI = -0.34–0.81, %difference = 25.7%) and non-athletes (*p* < 0.001, r = 0.70, 95% CI = 0.45–0.85, %difference = 49.9%). The nACSA of VL was significantly greater in the rugby players than in the sprinters (*p* = 0.001, r = 0.64, 95% CI = 0.35–0.82, %difference = 17.3%) and non-athletes (*p* < 0.001, r = 0.78, 95% CI = 0.58–0.89, %difference = 39.5%). This nACSA was also significantly greater in the sprinters than in the non-athletes (*p* = 0.024, r = 0.43, 95% CI = 0.06–0.69, %difference = 15.5%). The nACSA of VI was also significantly greater in the rugby players than in the sprinters (*p* = 0.002, r = 0.60, 95% CI = 0.29–0.80, %difference = 19.5%) and non-athletes (*p* < 0.001, r = 0.70, 95% CI = 0.43–0.85, %difference = 30.7%).

**Fig 4:**
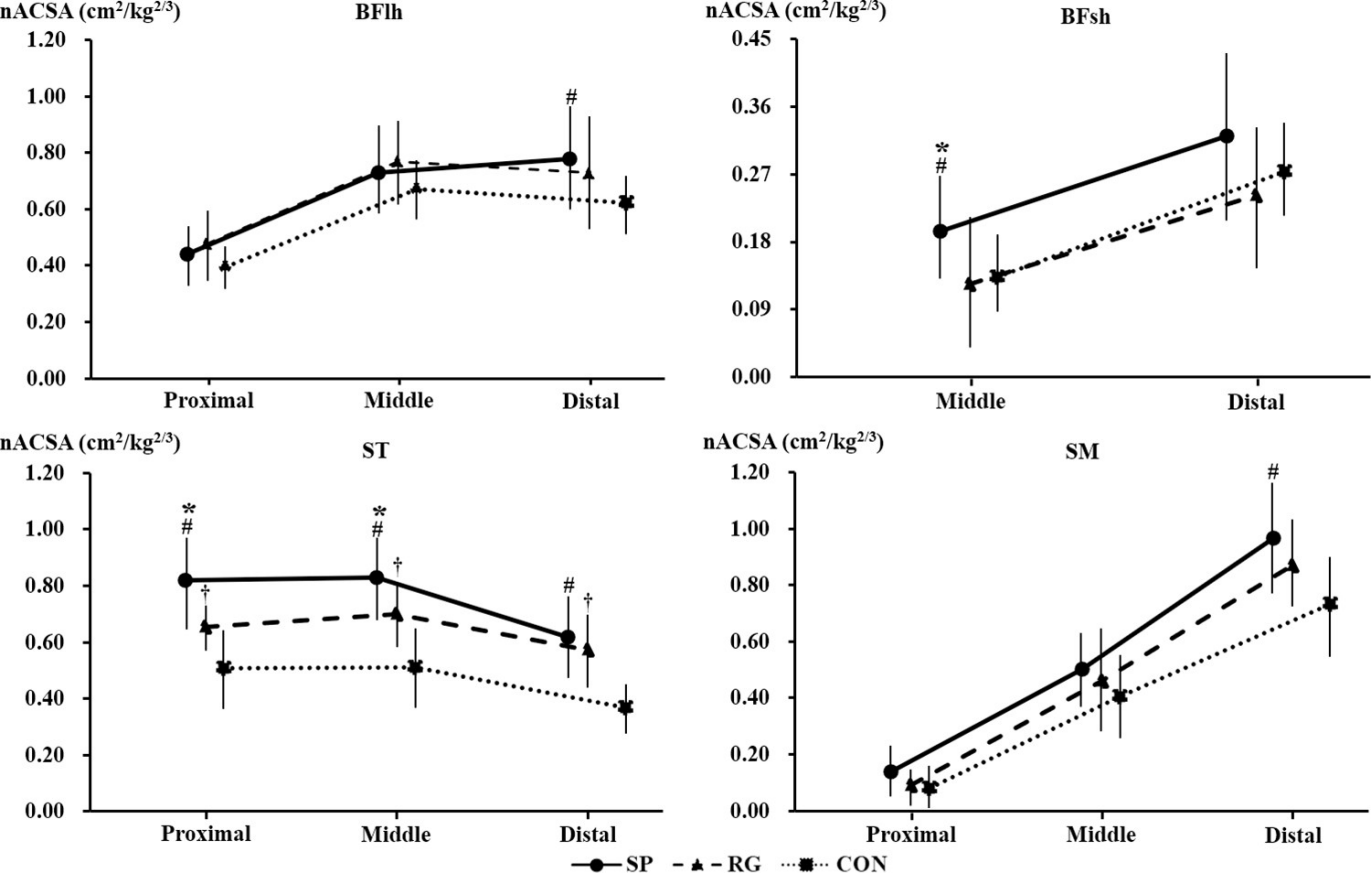
The normalized anatomical cross-sectional area in each region of the biceps femoris long head (BFlh), the biceps femoris short head (BFsh), the semitendinosus (ST), and the semimembranosus (SM) in the sprinters (SP), rugby players (RG), and non-athletes (CON). *Significant difference between the sprinters and the rugby players. #Significant difference between the sprinters and the non-athletes. †Significant difference between the rugby players and the non-athletes.

The significant participants’ group differences in nACSA were observed in the distal region of BFlh (*p* = 0.031, r = 0.334, 95% CI = 0.03–0.58), the middle region of BFsh (*p* = 0.009, r = 0.40, 95% CI = 0.11–0.63), all regions of ST (all *p* < 0.05, r = 0.64–0.67, 95% CI = 0.42–0.79), and the distal region of SM (*p* = 0.018, r = 0.37, 95% CI = 0.07–0.60), but not in the other regions (all *p* > 0.05, r = 0.12–0.31, 95% CI = -0.16–0.51) (Fig 4).

**Fig 5:**
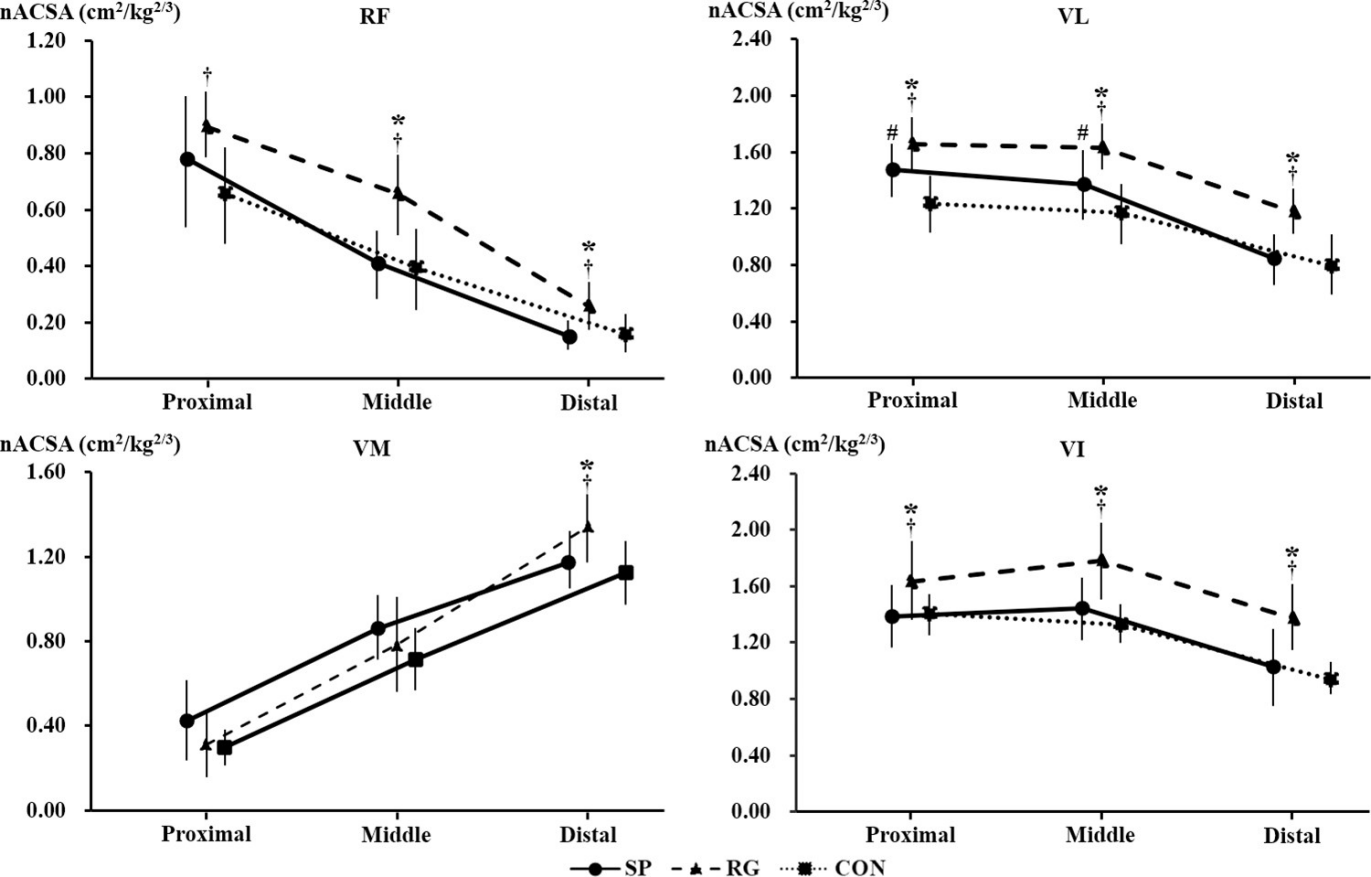
The normalized anatomical cross-sectional area in each region of the rectus femoris (RF), the vastus lateralis (VL), the vastus medialis (VM), and the vastus intermedius (VI) in the sprinters (SP), rugby players (RG), and non-athletes (CON). *Significant difference between the sprinters and the rugby players. #Significant difference between the sprinters and the non-athletes. †Significant difference between the rugby players and the non-athletes.

There were significant participants’ differences in nACSA for all regions of RF (all *p* < 0.05, r = 0.46–0.62, 95% CI = 0.18–0.78), VL (all *p* < 0.001, r = 0.60–0.61, 95% CI = 0.37–0.77), VM (*p* < 0.001, r = 0.54, 95% CI = 0.29–0.72), and VI (all *p* = 0.05, r = 0.42–0.58, 95% CI = 0.13–0.75) except for the proximal (*p* = 0.003, r = 0.46, 95% CI = 0.18–0.67) and middle regions (*p* = 0.162, r = 0.22, 95% CI = -0.10–0.49) of VM (Fig 5).

## Discussion

The main findings of this study were that nACSAs of BFsh and ST were significantly greater in the sprinters than in the rugby players whereas those of RF, VL and VI were significantly greater in the rugby players than in the sprinters. These results partly support our first hypothesis that sprinters possess greater ST and RF muscle sizes than rugby players. Moreover, region-specific nACSA differences between the athletes were observed within BFsh, ST, and RF, partially supporting our second assumption that sprinters and rugby players have different size distribution within the individual thigh muscles. To our knowledge, there is no information regarding the morphological differences of the individual thigh muscles between both athletes. The present study is the first to demonstrate that sub-elite sprinters and rugby players have unique morphological characteristics of the thigh muscles.

The present results suggest that sprinters, who can generate a higher running speed than other athletes [3], have greater BFsh and ST sizes than rugby players. In terms of muscle architecture, ST had the longest fascicle length among the hamstring muscles in a previous study [18]. Thus, ST are more suitable for generating high contraction velocities than BFlh, BFsh, and SM during sprint running [19]. In addition, electromyographic studies have demonstrated that the activation of ST was higher during knee flexion tasks (e.g., Nordic hamstring) than during hip extension tasks (e.g., stiff-leg deadlift) [20]. In fact, during the middle swing phase of the sprint running cycle in which the knee joint is more flexed, ST is preferentially activated compared with BFlh [21]. These findings imply that ST mainly acts in knee flexion during sprint running. Hence, the repetition of high-velocity knee flexion movements during sprinting might induce preferential hypertrophy of ST in sprinters. Regarding the preferential hypertrophy of BFsh in sprinters, recent research reported that muscle volume of BFsh (together with fiber typology of gastrocnemius medialis) could explain ~59% of the theoretical maximal horizontal velocity derived from linear 30-m sprints [22]. This finding implies that BFsh greatly contributes to the generation of horizontal velocity during acceleration phase of sprint running (e.g., approximately 0–30 m). Therefore, faster sprint running which demands greater horizontal velocity during acceleration phase could put an additional mechanical load on BFsh, and such repetitive sprinting activity may induce the specific hypertrophy of BFsh in sprinters.

Regarding the monoarticular vastus muscles of the quadriceps femoris, the sizes of VL and VI were greater in the rugby players than in the sprinters although that of VM did not differ between the two athlete groups. In competitive activities, rugby players frequently participate in contact actions. During the actions, the monoarticular vastus muscles need to generate a large knee extension torque to push the ground powerfully. In the quadriceps femoris, VL and VI possess a large potential for the generation of knee extension torque because these muscles have larger physiological cross-sectional areas than VM [23]. This notion is supported by a previous study that reported that changes in transverse relaxation time (indirect index of muscle activation) were significantly greater in VI than in VM after a bout of knee extension task [24]. Thus, the repeated high-intensity actions of knee extension during rugby competitive activities may be associated with the greater sizes of VL and VI in rugby players.

In addition to VL and VI, RF size was also smaller in the sprinters than in the rugby players in the present study. This finding is inconsistent with our hypothesis that muscle size of RF is larger in sprinters than in rugby players. Recent research indicated that muscle volumes of RF and VL could explain ~43% of the theoretical maximal vertical force derived from jumping and suggest that muscle sizes of RF and VL significantly contribute to a generation of a vertical force at low angular velocity [22]. This finding allows us to speculate that the preferential hypertrophy of RF and VL in rugby players may be caused by competitive actions that require a generation of a large vertical force at low movement velocity rather than the actions that require a production of a large horizontal force at high movement velocity like sprint running. More studies are needed to clarify the relationship between muscle hypertrophic pattern and rugby competitive activity.

In this study, the sprinters had greater nACSAs than the rugby players in the middle region of BFsh and the proximal-middle regions of ST whereas the rugby players possessed larger nACSA than the sprinters in the middle-distal regions of RF. Also, in all regions of VL and VI, nACSA was greater in the rugby players than in the sprinters. These results imply that sprinters and rugby players have different size distributions, especially within BFsh, ST, and RF. It has been demonstrated that each of ST and RF is separated into the proximal and distal regions because of its multiple motor nerve branches [18,25], which possibly lead to different muscle activities between regions [26,27]. For example, within ST, the activity level was higher in the proximal region than in the distal region during knee flexion exercise (i.e., Nordic hamstring) while that was comparable between the regions during hip extension exercise (i.e., stiff-leg deadlift) [27]. These findings allow us to speculate that the proximal region of ST greatly contributes to performing knee flexion movements. Also, the distal region of RF showed a higher activity level than the proximal region during knee extension exercise, and vice versa during hip flexion exercise [26], which indicate regional difference in the functional role that the proximal and distal regions of RF mainly act for hip flexion and knee extension, respectively. The region-specific muscle activity during the training session was reported to be associated with nonuniform muscle hypertrophy after long-term training intervention [28]. Thus, repetition of competitive movements such as knee flexion (e.g., when swinging the leg during sprint running) and knee extension (e.g., when pushing the ground during a rugby match) may lead to different size distribution between athletes in ST and RF. Meanwhile, a previous study indicated a large inter-individual variability for region-specific activity patterns of ST during fast running [29]. Additionally, in BFsh, the degree of change in T2 value (index of muscle activity) was comparable among regions following each of knee flexion (i.e., Nordic hamstring) and hip extension exercises (i.e., hip extension conic pulley) [30]. Further investigation is warranted to clarify how region-specific activation patterns within the individual thigh muscles during competitive activities impact muscle size distribution along their lengths.

For interpreting the morphological difference between athletes, their performance levels are essential information. In the present study, nine of the 14 rugby players previously belonged to the national team whereas none of the sprinters had such an experience. However, a recent study demonstrated that muscle volume normalized to body weight did not differ between elite sprinters (n = 5; season’s best 100-m sprint time, 10.10 ± 0.07 s) and sub-elite sprinters (n = 26; season’s best 100-m sprint time, 10.80 ± 0.30 s) in the individual muscles of the hamstrings and quadriceps femoris [7]. Therefore, the different performance levels between athletes may have a little effect on the main findings of the present study although larger sample size of elite sprinters is required in further study. Meanwhile, in the previous study [7], elite sprinters had significantly larger normalized muscle volumes of the gluteus maximus, sartorius, and tensor fasciae latae muscle than sub-elite sprinters. These muscles should be examined, especially when comparing muscle morphologies between sprinters and other sports event’s athletes.

The findings of this study would be beneficial for rugby players in designing purposeful training regimens aimed at improving sprint performance. The results of the present study showed that the rugby players had smaller ST and BFsh muscle sizes than the sprinters. As mentioned earlier, BFsh and ST mainly work as knee flexors during sprint running [21]. For rugby players, increasing muscle sizes of BFsh and ST would be one of the effective approaches for enhancing their force-generating capacity and achieving a faster running speed in a linear sprint although sprint during rugby competition includes not only a linear sprint but also changing direction and tackle. It was demonstrated that knee dominant exercise (i.e., Nordic hamstring) could preferentially activate ST [20]. Besides, ST was suggested to be selectively activated at a longer muscle length during Nordic hamstring (i.e., with hips flexed to 90°) [31]. Thus, Nordic hamstring, especially at longer muscle length, may be a beneficial exercise for selectively strengthening ST. Notably, the rugby players had smaller muscle size in the proximal and middle regions of ST and the middle region of BFsh than the sprinters. The region-specific role within the individual hamstring muscles remains unknown [29]. However, the aforementioned results may imply that strengthening a specific region within a muscle, not only a whole muscle, is important for enhancing sprint performance. A few recent studies reported that knee flexion exercise (i.e., Nordic hamstring and slide leg curl) could selectively activate the proximal and middle regions of ST [27,32]. These findings may be helpful information for athletes and their coaches to design training regimens aimed at selectively strengthening a specific region within the muscle and consider regional muscle hypertrophy.

This study has some limitations. First, the training regimen in daily practice (e.g., training load and the number of sets and repetitions) might differ between the sprinters and rugby players although both types of athletes routinely conducted similar resistance training exercises (i.e., bench press, squat, and power clean) two times a week. Thus, the training regimens in daily practice may also influence the unique morphological characteristics of the athletes in addition to their daily competitive activities. Also, the rugby players in this study could be divided into seven forwards and seven backs based on their competitive experience. It is generally considered that forwards repeatedly perform contact actions such as scrums, rucks, and mauls whereas backs frequently participate in maximal speed actions such as sprinting and jumping. Thus, the morphological characteristics of the rugby players in this study may be represented differently when the ratio of rugby playing positions varies. Second, it was difficult to measure the sizes of the other muscles (e.g., adductor muscles) using extended-field-of-view ultrasonography. Third, the locations of maximal ACSAs in the individual muscles could not be considered when determining those ACSA measurement locations. Ideally, the measurement locations of ACSAs should be determined based on the muscle belly of the individual muscles. However, the tendons of the lateral and medial hamstrings were bulging from 70% to 100% of the thigh length in some participants. Hence, it was hard to measure ACSAs of the individual hamstring muscles at > 70% of the thigh length using extended-field-of-view ultrasonography. Moreover, we needed to complete the ACSA measurements in a limited time (approximately within 40 min) due to the time constraints of the athletes. Thus, we measured ACSAs of the individual muscles from a range of 35% to 65% of the thigh length. We believed that the ACSA measurement locations in this study reflect the muscle bellies of the individual hamstrings and quadriceps femoris because the largest ACSAs of the muscles were mainly observed in a range of 40% to 70% of the thigh length, except for BFsh and VM [33,34]. Next, muscle size was presented as ACSA, not as physiological cross-sectional area (PCSA). It is well known that ACSA is the area of the cross-section of a muscle perpendicular to its fibers whereas PCSA is that of a muscle perpendicular to its longitudinal axis. If muscle architectural parameters (e.g., pennation angle) are different among the participants, the present results of ACSA may not be consistent with the results of PCSA. Thus, to calculate PCSA, we attempted to measure fascicle length and pennation angle of the individual muscles in a pilot study. However, we could not provide clear data on these architectural parameters because the directions of muscle fascicles were not identified clearly, especially in some parts of SM and VI. Meanwhile, it is known that BFsh and ST are fusiform muscles that have a smaller pennation angle than the other hamstring muscles [35]. Thus, the present results of BFsh and ST would also be observed when considering PCSA although the aforementioned parameters should be determined to compare muscle size between the athletes of the different sports events in the further study. We believe that the architecture of the individual muscles would be identified accurately by a promising technique (i.e., diffusion tensor imaging), which can image and assemble three-dimensional representations of muscle structure [36]. Besides, the normalization procedure for ACSA may affect the present results. In this study, we used the two-thirds power of the body mass for the ACSA normalization according to the theoretical dimension of ASCA. Meanwhile, some results were different from the present study when ACSAs were normalized by the one power of the body mass (S1 Fig). This suggests that the choice of the normalization procedure for ACSA is vital for interpreting the morphological characteristics. However, no consensus has been reached regarding the ideal normalization procedure for ACSA, which should be discussed in the future study. Finally, the main findings of the present study may not generalize to the other sports athletes (e.g., soccer and baseball).

In conclusion, the sizes of BFsh and ST were greater in sprinters than in rugby players and/or non-athletes whereas those of RF, VL, and VI were the greatest in the rugby players. Moreover, region-specific differences in muscle sizes between both athletes were observed within BFsh, ST, and RF. These results suggest that 1) sub-elite sprinters possess large BFsh and ST muscles, whereas rugby players have larger RF, VL, and VI muscles, and 2) sprinters and rugby players have different size distributions, especially within BFsh, ST, and RF. These findings would be beneficial to rugby players trying to enhance their sprint performance.

## Supporting information

S1 Fig The anatomical cross-sectional area normalized by the one power of the body mass in the examined muscles in the sprinters, rugby players, and non-athletes. (PDF)

S1 Table The absolute values of the anatomical cross-sectional area of each region of the individual muscles in the hamstrings and the quadriceps femoris in the sub-elite sprinters, rugby players, and non-athletes. (PDF)

S2 Table The absolute values of the anatomical cross-sectional area of the individual muscles and muscle groups in the hamstrings and the quadriceps femoris in the sub-elite sprinters, rugby players, and non-athletes. (PDF)
