# Supplementary material for: Sub-elite sprinters and rugby players possess different morphological characteristics of the individual hamstrings and quadriceps muscles
Source: PLoS One. 2021 Oct 26;16(10):e0259039. doi: 10.1371/journal.pone.0259039 (PMC8547647; doi:10.1371/journal.pone.0259039)
Supplement: S1 Fig — (PDF) [file pone.0259039.s001.pdf]

Additional results

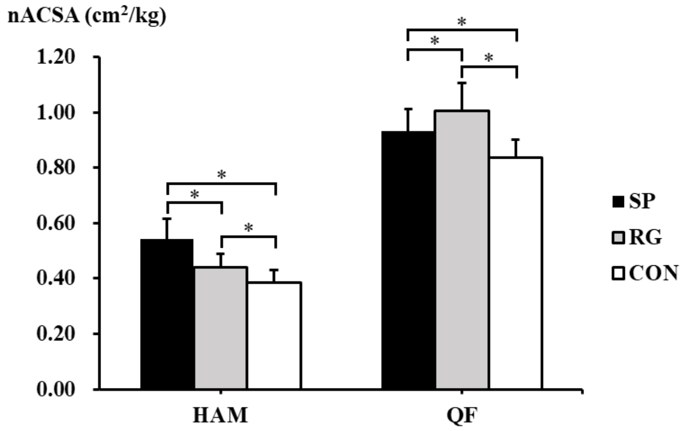

Fig A. The anatomical cross-sectional area normalized by the one power of the body mass in the hamstrings and quadriceps femoris in the sprinters (SP), rugby players (RG), and non-athletes (CON). *Significant difference between the groups. HAM, hamstrings; QF, quadriceps femoris.

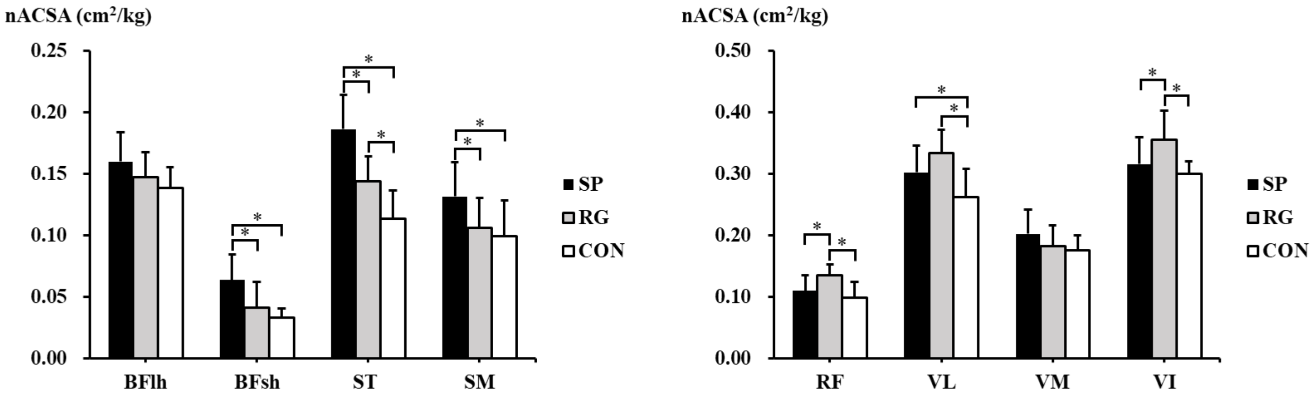

Fig B. The anatomical cross-sectional area normalized by the one power of the body mass in the individual muscles of the hamstrings and quadriceps femoris in the sprinters (SP), rugby players (RG), and non-athletes (CON). *Significant difference between the groups. BFlh, biceps femoris long head; BFsh, biceps femoris short head; ST, semitendinosus; SM, semimembranosus; RF, rectus femoris; VL, vastus lateralis; VM, vastus medialis; VI, vastus intermedius.

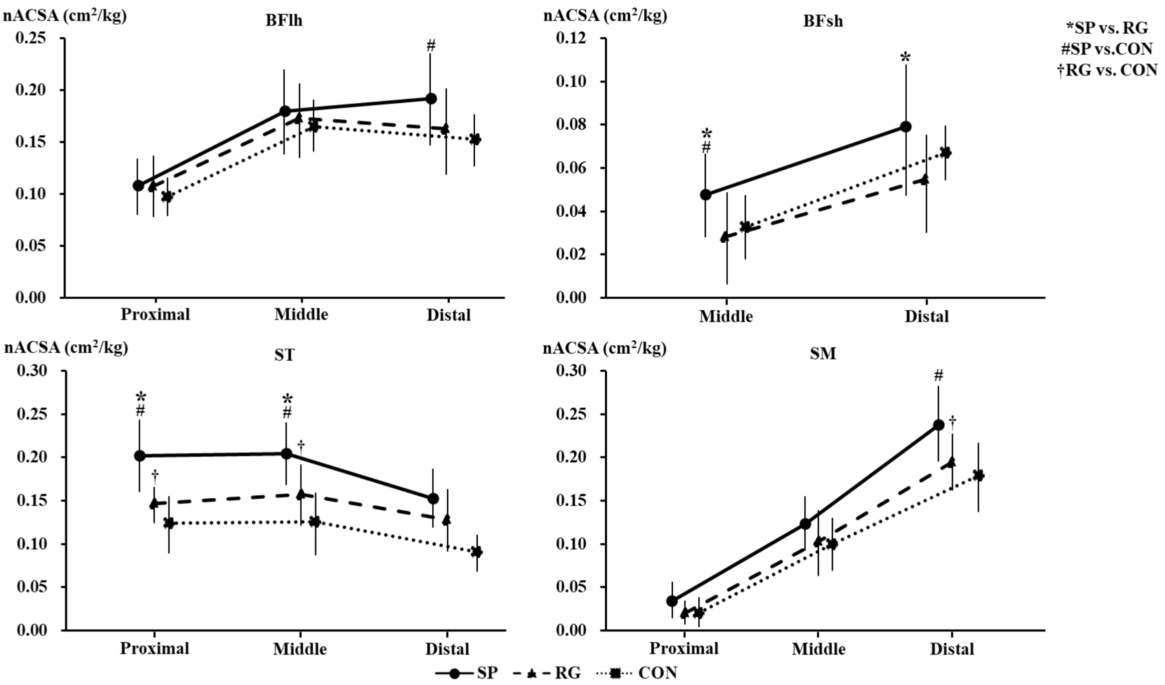

Fig C. The anatomical cross-sectional area normalized by the one power of the body mass in each region of the biceps femoris long head (BFlh), the biceps femoris short head (BFsh), the semitendinosus (ST), and the semimembranosus (SM) in the sprinters (SP), rugby players (RG), and non-athletes (CON). *Significant difference between the sprinters and the rugby players. #Significant difference between the sprinters and the non-athletes. †Significant difference between the rugby players and the non-athletes.

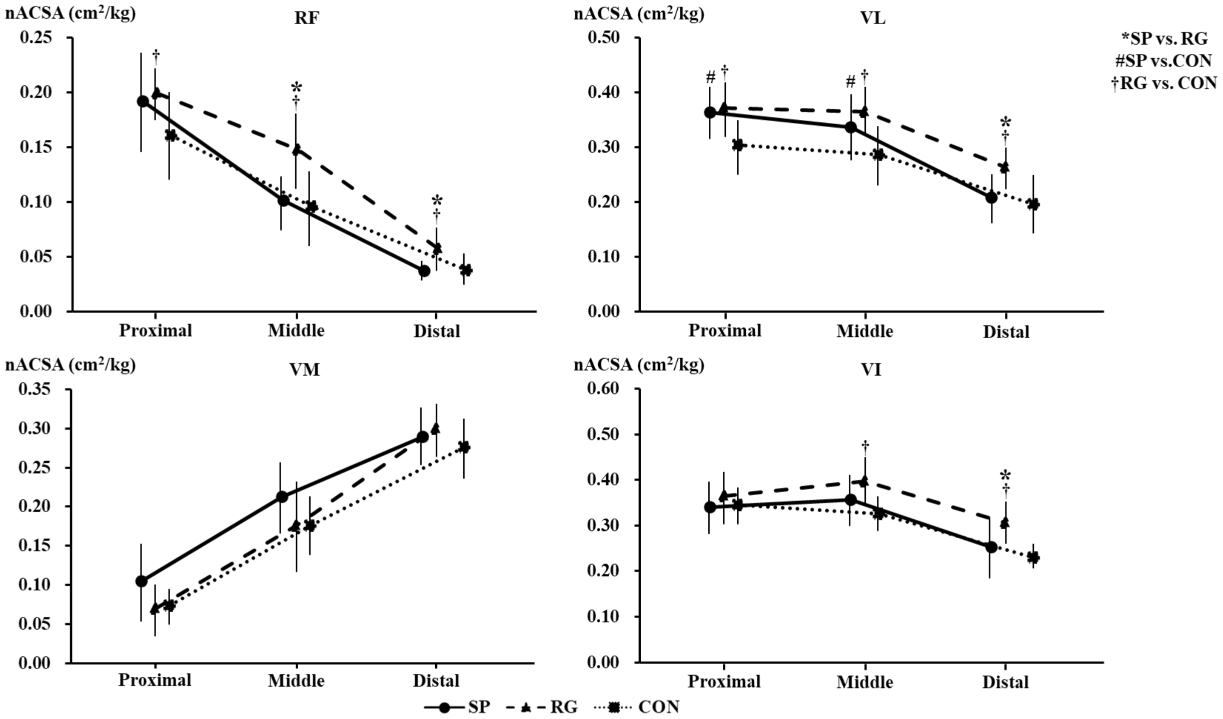

Fig D. The anatomical cross-sectional area normalized by the one power of the body mass in each region of the rectus femoris (RF), the vastus lateralis (VL), the vastus medialis (VM), and the vastus intermedius (VI) in the sprinters (SP), rugby players (RG), and non-athletes (CON). *Significant difference between the sprinters and the rugby players. #Significant difference between the sprinters and the non-athletes. †Significant difference between the rugby players and the non-athletes.
