# Supplementary material for: Sub-elite sprinters and rugby players possess different morphological characteristics of the individual hamstrings and quadriceps muscles
Source: PLoS One. 2021 Oct 26;16(10):e0259039. doi: 10.1371/journal.pone.0259039 (PMC8547647; doi:10.1371/journal.pone.0259039)
Supplement: S1 Table — (PDF) [file pone.0259039.s002.pdf]

Absolute values of the anatomical cross-sectional areas in the hamstrings (cm^2)

		Proximal				Middle				Distal			
		BFlh	BFsh	ST	SM	BFlh	BFsh	ST	SM	BFlh	BFsh	ST	SM
Sprinters	1	4.81	-	9.28	0.10	7.61	2.00	12.90	5.50	9.79	3.69	6.96	12.90
	2	6.95	-	15.49	0.93	12.44	3.58	14.16	6.29	12.88	3.44	9.99	12.38
	3	6.28	-	12.35	3.73	15.06	3.04	9.71	7.51	10.33	6.07	7.95	12.84
	4	6.51	-	14.94	1.78	9.15	0.67	15.97	4.59	13.31	4.78	13.44	11.34
	5	4.63	-	15.73	1.31	12.49	4.00	15.70	10.59	19.08	2.95	13.69	16.37
	6	7.09	-	9.33	3.64	9.87	5.34	14.13	9.71	15.95	5.37	9.77	12.44
	7	8.59	-	12.56	5.00	14.62	4.24	11.02	12.83	15.08	5.75	14.58	17.30
	8	7.36	-	10.92	0.76	12.94	3.17	11.53	7.75	9.00	4.06	8.84	13.70
	9	5.41	-	15.43	3.65	8.13	1.95	15.15	8.11	14.21	4.40	11.05	12.78
	10	11.03	-	15.97	5.03	13.11	3.89	15.70	11.69	14.90	6.70	10.59	19.99
	11	6.20	-	15.94	2.27	13.88	1.58	17.31	4.32	11.83	3.92	9.23	22.83
	12	8.34	-	11.26	0.70	12.40	4.37	10.62	10.90	8.08	7.37	10.57	18.46
	13	9.31	-	12.61	0.22	12.52	3.06	14.05	7.33	14.92	4.85	8.63	23.07
	14	9.08	-	17.28	2.23	13.70	3.89	13.21	8.94	10.42	10.42	7.68	17.07
	**Mean**	**7.26**	-	**13.51**	**2.24**	**11.99**	**3.20**	**13.65**	**8.29**	**12.84**	**5.27**	**10.21**	**15.96**
	**SD**	**1.83**	-	**2.64**	**1.70**	**2.36**	**1.27**	**2.27**	**2.63**	**3.09**	**1.95**	**2.33**	**3.96**

		Proximal				Middle				Distal			
		BFlh	BFsh	ST	SM	BFlh	BFsh	ST	SM	BFlh	BFsh	ST	SM
Rugby players	1	10.40	-	13.78	3.56	19.94	2.28	10.32	16.45	16.87	4.75	11.91	23.24
	2	8.63	-	12.74	1.42	13.98	1.09	15.48	4.09	26.70	2.92	9.96	14.98
	3	6.86	-	13.62	2.33	16.60	2.95	16.83	13.98	17.92	3.93	11.33	23.48
	4	10.91	-	15.06	1.40	14.58	1.95	15.85	10.21	14.60	5.05	8.56	15.05
	5	14.41	-	13.99	0.85	13.50	2.31	13.52	9.54	19.98	4.91	9.62	15.98
	6	10.24	-	13.97	3.70	20.07	3.05	11.82	14.63	14.77	4.52	16.33	20.37
	7	10.81	-	11.51	0.22	14.49	2.17	14.99	5.18	13.92	4.10	8.66	17.47
	8	8.12	-	17.00	0.50	20.13	1.86	12.86	7.62	13.18	2.28	12.70	22.46
	9	7.95	-	16.31	1.66	10.68	1.52	16.29	8.45	13.52	6.05	17.49	15.39
	10	11.96	-	12.69	0.65	11.73	2.52	15.63	6.23	9.86	5.34	10.97	11.14
	11	7.58	-	12.88	1.75	18.49	1.14	14.26	8.34	11.33	3.60	13.18	13.41
	12	7.83	-	11.14	0.55	12.04	1.62	13.77	3.64	8.83	4.09	10.55	19.10
	13	6.37	-	9.99	3.89	12.97	2.46	11.32	11.17	13.07	6.61	9.78	15.63
	14	10.72	-	9.12	2.27	16.13	6.86	11.70	9.60	10.92	7.82	8.79	16.17
	**Mean**	**9.48**	-	**13.13**	**1.77**	**15.38**	**2.41**	**13.90**	**9.22**	**14.68**	**4.71**	**11.42**	**17.42**
	**SD**	**2.24**	-	**2.22**	**1.24**	**3.24**	**1.41**	**2.05**	**3.88**	**4.62**	**1.45**	**2.74**	**3.78**

		Proximal				Middle				Distal			
		BFlh	BFsh	ST	SM	BFlh	BFsh	ST	SM	BFlh	BFsh	ST	SM
Non-athletes	1	6.54	-	9.70	0.90	11.46	2.57	6.31	8.85	10.00	4.54	5.34	14.36
	2	7.37	-	9.54	2.63	11.88	3.77	9.53	11.54	12.63	5.08	4.97	18.11
	3	7.11	-	8.15	0.81	12.65	3.38	11.60	7.24	11.15	4.54	5.21	10.86
	4	7.62	-	10.51	2.22	12.60	2.48	9.22	7.57	8.10	6.29	4.36	15.54
	5	8.71	-	6.16	4.25	12.42	2.68	6.08	8.53	9.48	5.83	4.10	15.20
	6	6.99	-	8.47	0.40	9.73	0.88	7.55	6.53	9.39	4.75	6.50	13.30
	7	6.97	-	6.78	1.66	11.46	2.33	8.95	5.90	14.50	4.99	8.09	12.01
	8	5.55	-	8.07	0.57	8.99	2.60	10.01	3.56	9.94	4.27	8.98	8.62
	9	4.91	-	6.80	0.80	9.30	0.63	7.23	6.52	8.85	4.57	5.47	13.68
	10	4.47	-	7.59	0.13	7.39	1.41	8.43	3.35	10.55	2.37	6.70	6.60
	11	5.41	-	15.23	0.64	12.10	2.92	14.56	6.28	9.17	5.79	7.05	12.42
	12	7.40	-	10.68	1.05	14.61	2.29	7.12	6.78	12.72	4.59	8.79	10.33
	13	5.39	-	4.04	0.14	8.57	1.66	4.92	2.72	6.75	2.52	4.62	8.45
	14	7.60	-	6.79	2.75	13.52	1.56	7.28	9.40	11.28	3.68	5.61	10.82
	**Mean**	**6.57**	-	**8.47**	**1.35**	**11.19**	**2.23**	**8.49**	**6.77**	**10.32**	**4.56**	**6.13**	**12.16**
	**SD**	**1.23**	-	**2.66**	**1.20**	**2.08**	**0.90**	**2.48**	**2.44**	**2.02**	**1.13**	**1.60**	**3.15**

Absolute values of the anatomical cross-sectional areas in the quadriceps femoris (cm^2)

		Proximal				Middle				Distal			
		RF	VL	VM	VI	RF	VL	VM	VI	RF	VL	VM	VI
Sprinters	1	7.95	18.34	6.15	17.53	8.07	12.97	13.76	20.61	1.43	12.22	18.37	12.74
	2	9.08	27.38	7.95	19.37	6.31	23.83	12.93	23.57	4.07	16.76	18.79	14.50
	3	8.77	19.73	13.94	18.27	4.49	14.34	18.47	14.39	1.91	8.85	19.28	16.09
	4	16.46	20.44	5.35	26.70	5.39	27.64	10.79	27.73	2.82	17.72	15.35	23.98
	5	10.08	22.14	5.54	23.09	4.02	23.35	14.46	29.18	2.19	14.68	18.10	19.63
	6	11.82	26.58	6.95	20.45	6.00	22.80	15.34	24.77	2.29	12.25	20.16	15.10
	7	13.29	24.96	6.99	21.38	8.11	23.72	13.45	24.56	2.87	13.37	21.93	20.21
	8	23.05	27.47	5.02	26.19	9.37	29.79	12.48	23.61	3.01	19.36	22.54	17.14
	9	13.22	26.80	3.20	18.45	5.60	24.58	12.83	24.69	2.91	16.00	13.99	27.18
	10	14.13	26.76	5.85	24.11	6.16	26.49	14.80	20.66	1.33	12.49	20.08	11.56
	11	15.23	28.95	4.24	27.83	10.14	25.17	14.27	28.47	2.74	17.84	19.79	15.11
	12	7.11	24.90	10.47	19.78	3.80	21.46	17.44	20.58	2.18	11.45	21.00	10.95
	13	16.09	22.44	7.75	30.86	9.53	21.05	12.58	25.56	3.16	10.70	21.74	15.05
	14	15.43	23.42	6.83	26.01	8.10	20.10	14.13	25.99	2.18	12.01	19.92	17.59
	**Mean**	**12.98**	**24.31**	**6.87**	**22.86**	**6.79**	**22.66**	**14.12**	**23.88**	**2.51**	**13.98**	**19.36**	**16.92**
	**SD**	**4.28**	**3.28**	**2.69**	**4.16**	**2.09**	**4.62**	**2.00**	**3.88**	**0.73**	**3.12**	**2.39**	**4.58**

		Proximal				Middle				Distal			
		RF	VL	VM	VI	RF	VL	VM	VI	RF	VL	VM	VI
Rugby players	1	15.39	30.29	3.74	32.99	11.65	34.59	22.03	38.48	4.38	20.60	26.30	25.15
	2	17.64	32.55	2.98	31.54	15.17	35.51	11.15	42.03	8.44	23.92	27.81	32.75
	3	18.07	39.99	4.38	34.77	16.39	33.76	10.86	42.05	7.09	25.54	29.83	31.86
	4	18.49	33.27	5.97	38.05	17.70	31.82	10.55	42.25	5.81	27.12	31.81	30.93
	5	21.19	36.19	6.94	40.33	15.52	33.27	13.40	42.78	4.82	26.69	30.81	30.61
	6	23.72	40.56	7.68	42.23	13.11	34.74	16.18	43.18	3.72	26.36	29.79	30.45
	7	19.82	35.81	7.16	43.19	13.37	39.51	16.68	41.71	6.24	27.47	31.18	34.61
	8	25.02	37.01	10.31	36.52	12.94	38.03	20.71	44.20	2.37	27.20	32.30	40.07
	9	16.95	25.04	3.06	30.60	10.08	31.80	17.34	30.72	5.79	21.24	27.47	23.60
	10	12.10	25.86	3.12	25.96	9.37	23.20	9.90	23.55	3.25	14.98	15.63	17.27
	11	16.63	34.90	3.52	28.14	18.02	29.66	11.14	27.40	6.90	25.64	22.67	24.20
	12	15.37	30.83	9.08	31.32	9.11	30.16	20.24	32.63	4.25	20.96	23.86	29.01
	13	15.80	30.72	10.75	17.15	11.04	26.90	16.12	22.39	3.61	18.88	22.60	16.74
	14	15.19	30.44	7.82	26.27	10.95	33.16	20.62	30.41	4.71	22.92	25.51	21.20
	**Mean**	**17.96**	**33.11**	**6.18**	**32.79**	**13.17**	**32.58**	**15.49**	**35.98**	**5.10**	**23.54**	**26.97**	**27.75**
	**SD**	**3.51**	**4.66**	**2.75**	**7.14**	**2.99**	**4.23**	**4.33**	**7.85**	**1.69**	**3.76**	**4.64**	**6.67**

		Proximal				Middle				Distal			
		RF	VL	VM	VI	RF	VL	VM	VI	RF	VL	VM	VI
Non-athletes	1	11.31	20.73	4.35	21.62	12.70	19.96	9.77	20.29	3.12	14.22	23.49	13.43
	2	12.47	22.33	4.70	27.35	6.94	21.74	16.58	21.15	2.81	16.62	21.98	19.68
	3	12.31	17.48	6.18	22.29	6.92	15.21	11.98	22.04	3.64	11.39	14.90	15.72
	4	7.49	16.49	3.93	28.37	5.61	18.70	14.76	19.44	2.00	12.71	17.31	14.73
	5	10.03	18.03	6.97	20.72	4.35	16.91	13.63	21.15	1.99	11.65	19.85	12.62
	6	9.13	23.63	7.42	23.95	5.16	14.95	14.90	21.20	1.01	8.51	21.04	11.80
	7	20.08	25.65	5.24	27.26	11.37	23.92	9.31	24.63	4.78	19.02	21.14	18.85
	8	9.68	15.78	4.52	24.53	7.22	16.38	12.58	22.93	1.80	8.35	16.92	16.03
	9	10.19	20.17	6.06	21.34	4.53	23.72	13.30	22.66	2.25	17.18	17.61	15.80
	10	6.04	13.62	3.57	21.56	1.74	13.09	8.66	17.06	1.60	10.54	13.02	14.43
	11	12.32	23.50	4.97	23.44	8.28	20.15	10.43	28.42	3.93	8.35	21.04	18.97
	12	11.81	25.42	3.52	21.88	7.06	20.79	12.11	26.67	4.25	15.32	16.67	17.63
	13	10.44	23.01	3.08	17.58	5.77	23.86	8.02	17.89	1.99	18.04	16.22	14.19
	14	9.99	21.88	4.34	23.57	5.01	21.66	10.18	22.47	1.55	12.65	19.88	14.24
	**Mean**	**10.95**	**20.55**	**4.92**	**23.25**	**6.62**	**19.36**	**11.87**	**22.00**	**2.62**	**13.18**	**18.65**	**15.58**
	**SD**	**3.21**	**3.74**	**1.31**	**2.93**	**2.81**	**3.57**	**2.58**	**3.09**	**1.15**	**3.64**	**2.99**	**2.43**
