# Supplementary material for: Sub-elite sprinters and rugby players possess different morphological characteristics of the individual hamstrings and quadriceps muscles
Source: PLoS One. 2021 Oct 26;16(10):e0259039. doi: 10.1371/journal.pone.0259039 (PMC8547647; doi:10.1371/journal.pone.0259039)
Supplement: S2 Table — (PDF) [file pone.0259039.s003.pdf]

Absolute values of the anatomical cross-sectional areas in the hamstrings (cm^2)

		BFlh	BFsh	ST	SM	Muscle group
**Sprinters**	1	7.41	2.85	9.71	6.17	26.13
	2	10.76	3.51	13.21	6.53	34.01
	3	10.56	4.56	10.00	8.03	33.14
	4	9.66	2.73	14.78	5.90	33.07
	5	12.07	3.48	15.04	9.42	40.01
	6	10.97	5.35	11.08	8.60	36.00
	7	12.77	5.00	12.72	11.71	42.20
	8	9.77	3.62	10.43	7.40	31.22
	9	9.25	3.17	13.88	8.18	34.48
	10	13.01	5.30	14.09	12.24	44.63
	11	10.64	2.75	14.16	9.81	37.35
	12	9.61	5.87	10.81	10.02	36.31
	13	12.25	3.96	11.76	10.20	38.17
	14	11.07	7.15	12.72	9.41	40.36
	**Mean**	**10.70**	**4.23**	**12.46**	**8.83**	**36.22**
	**SD**	**1.52**	**1.34**	**1.82**	**1.94**	**4.78**

		BFlh	BFsh	ST	SM	Muscle group
**Rugby players**	1	15.74	3.52	12.00	14.42	45.67
	2	16.43	2.01	12.73	6.83	38.00
	3	13.79	3.44	13.92	13.26	44.42
	4	13.36	3.50	13.15	8.89	38.91
	5	15.97	3.61	12.38	8.79	40.74
	6	15.03	3.79	14.04	12.90	45.75
	7	13.07	3.13	11.72	7.62	35.55
	8	13.81	2.07	14.19	10.19	40.26
	9	10.72	3.78	16.70	8.50	39.70
	10	11.18	3.93	13.10	6.00	34.21
	11	12.47	2.37	13.44	7.83	36.10
	12	9.57	2.86	11.82	7.76	32.01
	13	10.80	4.53	10.36	10.23	35.93
	14	12.59	7.34	9.87	9.35	39.14
	**Mean**	**13.18**	**3.56**	**12.82**	**9.47**	**39.03**
	**SD**	**2.13**	**1.31**	**1.71**	**2.50**	**4.18**

		BFlh	BFsh	ST	SM	Muscle group
**Non-athletes**	1	9.34	2.37	7.12	8.03	26.86
	2	10.63	2.95	8.01	10.76	32.35
	3	10.30	2.64	8.32	6.30	27.57
	4	9.44	2.92	8.03	8.44	28.83
	5	10.20	2.84	5.45	9.33	27.81
	6	8.71	1.88	7.51	6.74	24.83
	7	10.98	2.44	7.94	6.53	27.88
	8	8.16	2.29	9.02	4.25	23.72
	9	7.69	1.73	6.50	7.00	22.92
	10	7.47	1.26	7.57	3.36	19.66
	11	8.89	2.90	12.28	6.45	30.52
	12	11.58	2.29	8.87	6.05	28.79
	13	6.90	1.39	4.53	3.77	16.60
	14	10.80	1.75	6.56	7.65	26.77
	**Mean**	**9.36**	**2.26**	**7.69**	**6.76**	**26.08**
	**SD**	**1.45**	**0.57**	**1.82**	**2.07**	**4.22**

Absolute values of the anatomical cross-sectional areas in the quadriceps femoris (cm2)

		RF	VL	VM	VI	Muscle group
**Sprinters**	1	5.82	14.51	12.76	16.96	50.05
	2	6.49	22.65	13.22	19.15	61.51
	3	5.05	14.31	17.23	16.25	52.84
	4	8.23	21.93	10.50	26.14	66.80
	5	5.43	20.06	12.70	23.96	62.15
	6	6.70	20.54	14.15	20.10	61.50
	7	8.09	20.68	14.12	22.05	64.94
	8	11.81	25.54	13.35	22.31	73.01
	9	7.25	22.46	10.01	23.44	63.15
	10	7.21	21.91	13.58	18.77	61.47
	11	9.37	23.99	12.76	23.80	69.92
	12	4.36	19.27	16.31	17.10	57.04
	13	9.59	18.07	14.02	23.82	65.50
	14	8.57	18.51	13.63	23.20	63.90
	**Mean**	**7.43**	**20.32**	**13.45**	**21.22**	**62.41**
	**SD**	**2.02**	**3.23**	**1.88**	**3.13**	**6.09**

		RF	VL	VM	VI	Muscle group
**Rugby players**	1	10.47	28.49	17.36	32.21	88.53
	2	13.75	30.66	13.98	35.44	93.83
	3	13.85	33.10	15.02	36.23	98.20
	4	14.00	30.74	16.11	37.08	97.92
	5	13.84	32.05	17.05	37.91	100.85
	6	13.52	33.89	17.88	38.62	103.91
	7	13.14	34.26	18.34	39.83	105.57
	8	13.44	34.08	21.10	40.26	108.89
	9	10.94	26.03	15.95	28.31	81.23
	10	8.24	21.35	9.55	22.26	61.40
	11	13.85	30.07	12.44	26.58	82.94
	12	9.58	27.32	17.73	30.99	85.61
	13	10.15	25.50	16.49	18.76	70.91
	14	10.28	28.84	17.98	25.96	83.07
	**Mean**	**12.08**	**29.74**	**16.21**	**32.17**	**90.20**
	**SD**	**2.01**	**3.78**	**2.83**	**6.87**	**13.68**

		RF	VL	VM	VI	Muscle group
**Non-athletes**	1	9.05	18.30	12.53	18.45	58.33
	2	7.41	20.23	14.42	22.73	64.79
	3	7.62	14.69	11.02	20.02	53.36
	4	5.03	15.97	12.00	20.84	53.85
	5	5.46	15.53	13.48	18.16	52.63
	6	5.10	15.70	14.45	18.98	54.23
	7	12.08	22.86	11.90	23.58	70.41
	8	6.23	13.50	11.34	21.16	52.23
	9	5.66	20.35	12.32	19.93	58.27
	10	3.13	12.42	8.41	17.68	41.64
	11	8.18	17.33	12.15	23.61	61.27
	12	7.70	20.51	10.77	22.06	61.04
	13	6.06	21.63	9.11	16.55	53.36
	14	5.52	18.73	11.46	20.09	55.80
	**Mean**	**6.73**	**17.70**	**11.81**	**20.28**	**56.51**
	**SD**	**2.18**	**3.18**	**1.72**	**2.19**	**6.78**
